# A Rare Case of an Incidental Near-Complete Gastric Band Erosion Without Perforation

**DOI:** 10.7759/cureus.25636

**Published:** 2022-06-03

**Authors:** Vincent Wong, Catherine Choi, He Qiu, Alexander Kaye, Weizheng Wang

**Affiliations:** 1 Internal Medicine/Pediatrics, Rutgers University New Jersey Medical School, Newark, USA; 2 Gastroenterology and Hepatology, Rutgers University, Newark, USA; 3 Gastroenterology and Hepatology, Rutgers University New Jersey Medical School, Newark, USA; 4 Internal Medicine, Rutgers University, Newark, USA

**Keywords:** laparoscopic adjustable gastric band, esophagogastroduodenoscopy (egd), endoscopic removal, effect of bariatric surgery, adjustable gastric band complications

## Abstract

Laparoscopic gastric banding has been favored for the treatment of morbid obesity because it is minimally invasive, effective, and reversible. One of the complications is gastric band erosion which can cause abdominal pain, hematemesis, and hematochezia. Erosions can be partial and can lead to intra-abdominal free air, peritonitis, and sepsis. Endoscopic removal of the gastric band can be done safely and effectively using a wire and a mechanical lithotripter. We describe a patient with a rare case of an incidental near-complete gastric band erosion without perforation and subsequent endoscopic removal.

## Introduction

Bariatric surgery has transitioned from an open surgical approach to a laparoscopic procedure for the treatment of morbid obesity [1,2]. Gastric banding was one of the most preferred when it was first introduced in 1993 because it is minimally invasive, effective, and reversible [3,4]. An inflatable band is placed laparoscopically below the gastro-esophageal junction to create a smaller gastric pouch [2]. The size of this pouch can then be adjusted via a subcutaneous port externally [2]. Unfortunately, there has been a recent decline in the popularity of this procedure due to its high complication rates of up to 40% [2,3]. We present a rare case of gastric band erosion without signs of gastric perforation.

## Case presentation

A 62-year-old female with a history of a laparoscopic gastric band placed ten years ago presented with melena, abdominal pain, and hematemesis. She also endorsed lightheadedness and heartburn but denied any episodes of syncope, fever, chest pain, or shortness of breath. She denied any recent illicit drug or alcohol use; however, she had been taking non-steroidal anti-inflammatory drugs (NSAIDs) for pain.

In the emergency department, her vitals showed tachycardia at 116 beats per minute. Her laboratory values (Table 1) were significant for anemia with hemoglobin of 8.3 g/dL. Chest X-ray did not show any abdominal free air. Her physical exam was notable for tachycardia, orthostasis, and melena on the digital rectal exam. Cardiac and pulmonary exams were normal, and her abdomen was soft, non-tender, and non-distended. There were no visible fissures, masses, or hemorrhoids on the external rectal exam. The patient was started on intravenous pantoprazole of 40 milligrams twice daily, and the gastroenterology team was consulted.

**Table 1:** Laboratory values on presentation

Test	Value	Normal
Hemoglobin	8.3 g/dL	14.0 - 18.0 g/dL
Platelets	189x10^3/uL	150 - 450 x10^3/uL
Blood urea nitrogen (BUN)	34 mg/dL	6 - 20 mg/dL
Creatinine	0.5 mg/dL	0.7 - 1.2 mg/dL
Lipase	21 u/L	13 - 60 u/L
Prothrombin time (PT)	14.7 seconds	12.1 - 14.8 seconds
International normalized ratio (INR)	1.1	0.9 - 1.17
Partial thromboplastin time (PTT)	24.7 seconds	24 - 34.2 seconds
Troponin	<0.01 ng/mL	0 - 0.30 mg/mL

**Figure 1:**
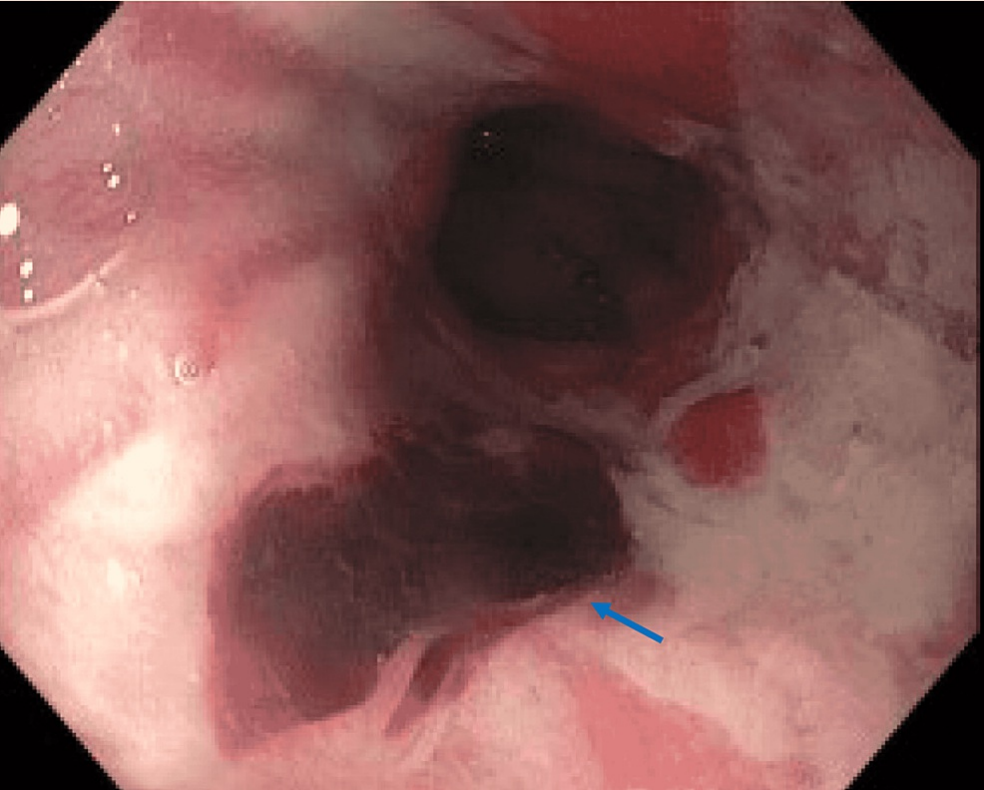
Gastroesophageal junction on esophagogastroduodenoscopy shows a Mallory Weiss tear with an adherent clot (blue arrow)

**Figure 2:**
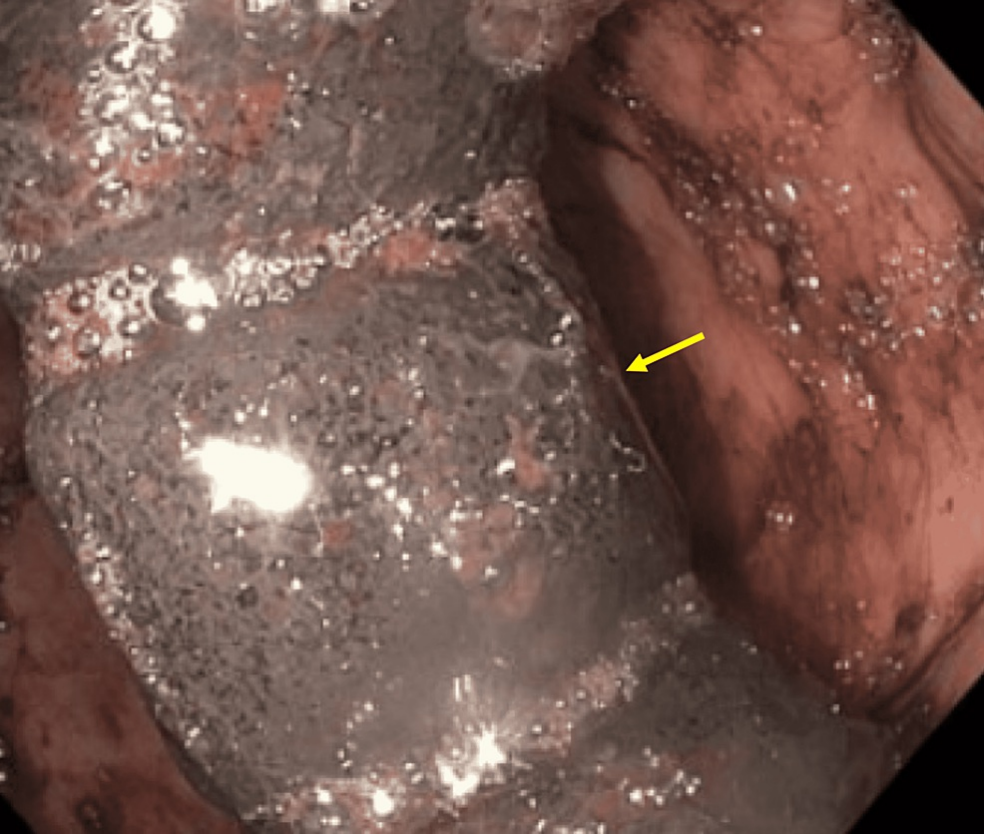
Gastric band (yellow arrow) visualized in the stomach lumen on esophagogastroduodenoscopy (EGD)

**Figure 3:**
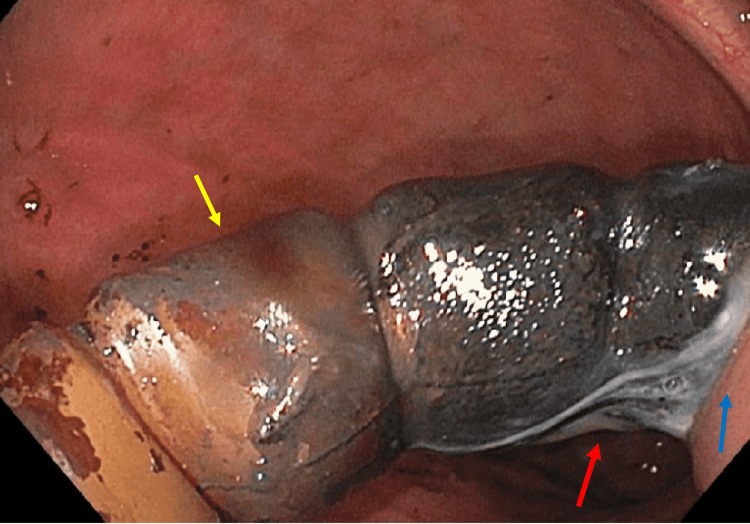
Gastric band (yellow arrow) visualized in the stomach lumen while attached to the gastric cardia wall (blue arrow) by white fibrinous material (red arrow)

Esophagogastroduodenoscopy (EGD) showed esophageal ulcers with esophagitis without active bleeding, a Mallory Weiss tear with clot at the gastroesophageal junction (Figure 1), a medium-sized hiatal hernia, chronic active gastritis at the gastric antrum and a gastric band eroded two-thirds into the gastric lumen (Figure 2). The remainder of the band was seen in the gastric cardia wall attached by fibrous tissue (Figure 3). No other active sites of bleeding were found.

**Figure 4:**
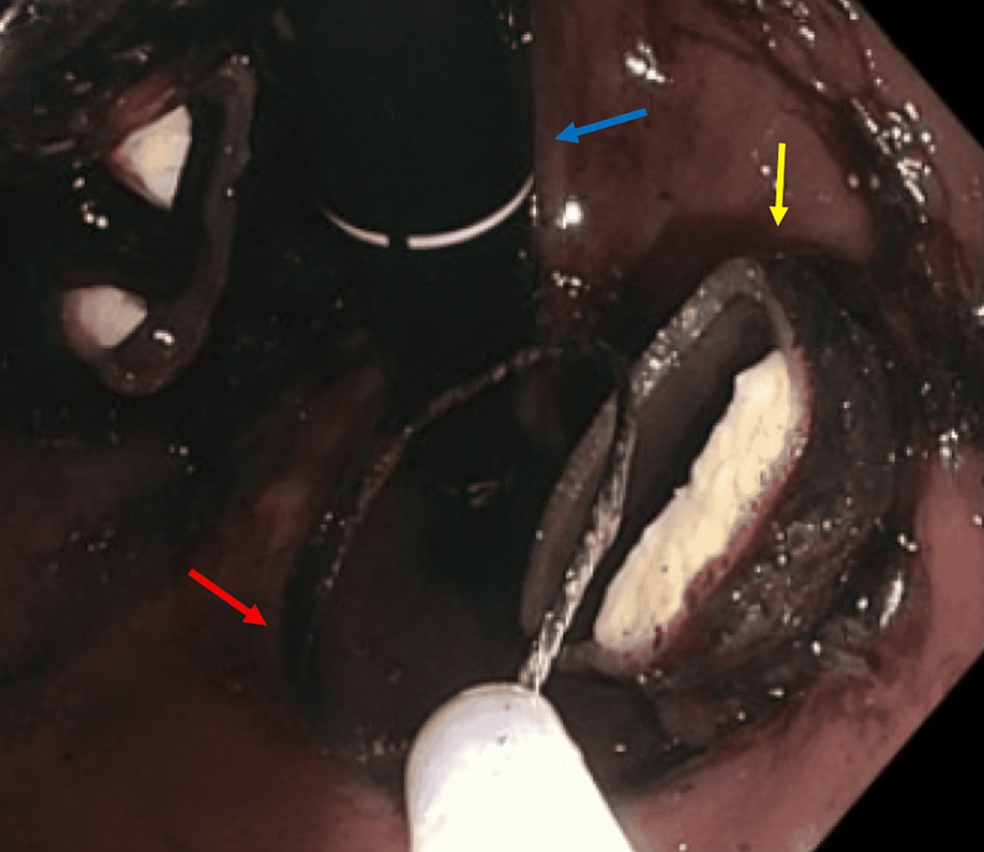
Transected gastric band (yellow arrow) with the snare (red arrow) seen on esophagogastroduodenoscopy EGD (scope - blue arrow)

**Figure 5:**
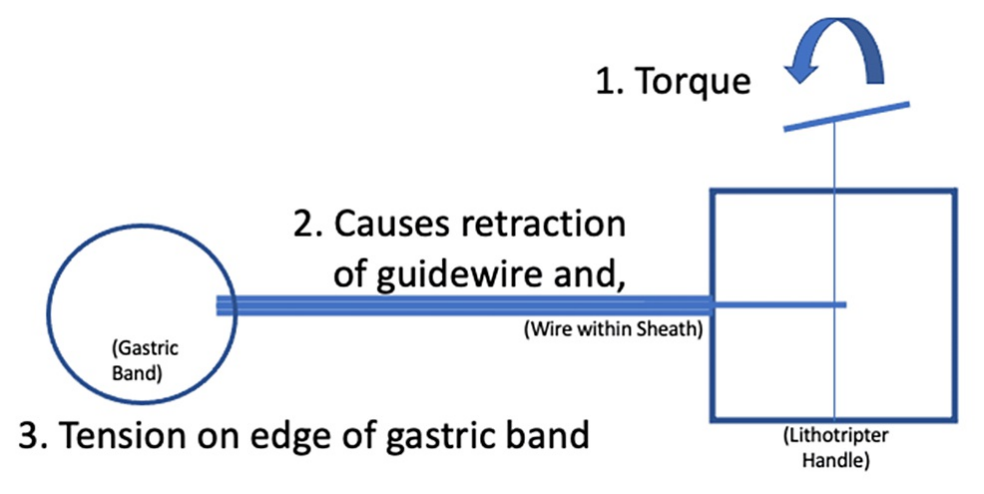
Diagram illustrating the technique used for endoscopic retrieval of the eroded gastric band

**Figure 6:**
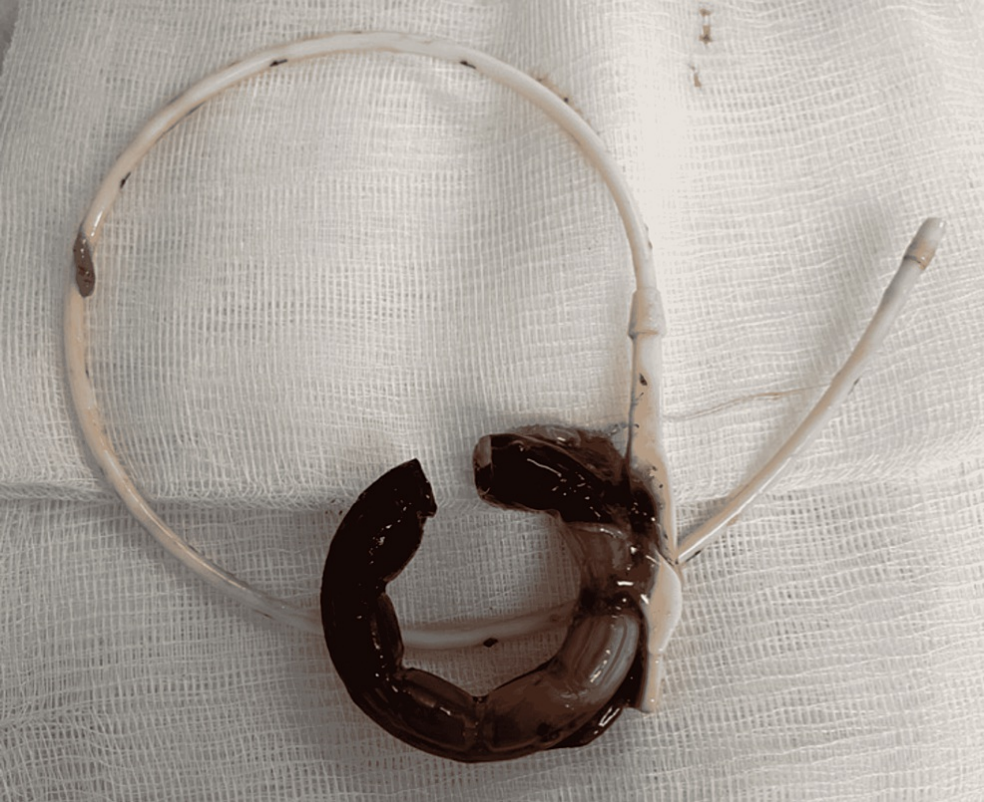
Transected gastric band after removal with the snare

There was no specific device available to remove the band, so the mechanical lithotripter was modified for this procedure. The 195-centimeter metallic-rubber sheath of the lithotripter (Olympus Medical Systems Corp., Tokyo, Japan) was cut by about 20 centimeters to accommodate the 0.64 millimeters in diameter Jagwire™ (Boston Scientific, Natick, US) that was approximately 450 centimeters in length. The wire was inserted into the double-channel endoscope to traverse the band, and the polypectomy snare (Boston Scientific, Natick, US) was used to grab the end of the wire until it came out of the patient's mouth. The modified lithotripter sheath was inserted into the endoscope's biopsy channel, and both ends of the wire were inserted through the sheath. The sheath and the endoscope were advanced into the stomach. The ends of the wire outside of the endoscope were inserted into the emergency lithotripter handle, and torque was applied until the gastric band was severed (Figures 4-5). The band was then removed with the snare (Figure 6). The entire stomach was visualized afterward without evidence of bleeding or perforation.

The patient was monitored after the procedure, no signs of emphysema were present, and her abdominal pain and hematemesis resolved. She was then discharged home and followed up in the clinic without any further symptoms.

## Discussion

Laparoscopic gastric banding rates have recently declined, from 42% in 2008 to 6% of bariatric cases in 2015, likely due to the number of complications [1,2,5]. The major complications include gastric band erosion (28%), port-tubing disconnection (20%), band slippage (4-13%), port infection (2%), and gastric perforation (0.1-0.8%) [2]. Patients generally require surgical revision or removal of the gastric band, but an endoscopic approach has been a safe and effective alternative [3,6]. 

Gastric band erosions can have a variable and unpredictable course, usually presenting several years post-placement [2]. Patients can be asymptomatic or can have nausea, vomiting, abdominal pain, hematemesis, or hematochezia [7]. Several mechanisms have been proposed for the causes of gastric band erosion, which is based on postoperative timing. In the early stage, it can be from iatrogenic gastric wall damage or micro-perforation after infection [2]. In the late stage, it can be secondary to chronic ischemia from gastric wall pressure or foreign body rejection, causing fibrous tissue formation and mural erosion [1,2]. At times, the gastric band is only partially eroded, allowing for the formation of intra-abdominal free air through the stomach, peritonitis, and sepsis [2,4,7,8]. 

Our patient was incidentally found to have the band eroded into her stomach. Her presenting symptoms were likely due to acid reflux from the failure of the band, NSAIDs use, and mucosal irritation from the band during respiration. These led to vomiting, esophagitis, formation of esophageal ulcers, and a Mallory Weiss tear. Fortunately, there were no endoscopically visible signs of active bleeding or perforation, and her symptoms resolved after removal.

We also demonstrated an endoscopic method of retrieval of the gastric band [3,4,6,9-12]. There is literature documenting the use of an endoscopic gastric band cutter; however, this device is not available in the United States [13]. Flor et al. developed a similar technique, which was done for our patient, using the endoscope, a wire, and the mechanical lithotripter where the band can be transected and broken for retrieval through the mouth [4,9,14]. There have been cases where argon plasma coagulation with 25 watts of energy and endoscissors were used to cut the band, but their success rates depend on the thickness of the band [10-12,15]. The modality of choice is based on the expertise of the operator. Pneumoperitoneum is a possible complication, so carbon dioxide is often used instead of air to mitigate abdominal distension, and band erosions of less than 50% into the stomach lumen are not immediately removed [7,10]. Patients can be subsequently discharged on the same day of the procedure or after 24 hours of observation [7,10,13-15]. For those who fail endoscopic retrieval, surgical removal is often indicated [10,16].

## Conclusions

Gastric bands are still an option for weight loss for patients who do not want extensive procedures. However, they should be informed about the possible complications, especially gastric band erosions. Routine follow-up after placement of the band is essential, and band erosion should be considered for patients with new-onset hematemesis, abdominal pain, and melena.
